# Integrated Analysis of Transcriptome and Metabolome Reveals New Insights into the Formation of Purple Leaf Veins and Leaf Edge Cracks in *Brassica juncea*

**DOI:** 10.3390/plants11172229

**Published:** 2022-08-28

**Authors:** Kaijing Zhang, Dekun Yang, Yuchao Hu, Martin Kagiki Njogu, Jingjing Qian, Li Jia, Congsheng Yan, Ziang Li, Xing Wang, Liping Wang

**Affiliations:** 1 College of Agriculture, Anhui Science and Technology University, Chuzhou 233100, China; 2 Department of Plant Science, Chuka University, Chuka P.O. Box 109-60400, Kenya; 3 Key Laboratory of Genetic Improvement and Ecophysiology of Horticultural Crop, Institute of Horticulture, Anhui Academy of Agricultural Sciences, Hefei 230001, China; 4 School of Landscape and Ecological Engineering, Hebei University of Engineering, Handan 056038, China

**Keywords:** *Brassica juncea*, purple leaf vein, leaf edge cracks, multiomics, regulation pathway

## Abstract

Purple leaf veins and leaf edge cracks comprise the typical leaf phenotype of *Brassica juncea*; however, the molecular mechanisms and metabolic pathways of the formation of purple leaf veins and leaf edge cracks remain unclear. In this study, transcriptome and metabolome analyses were conducted to explore the regulation pathway of purple leaf vein and leaf edge crack formation based on four mustard samples that showed different leaf colors and degrees of cracking. The results showed genes with higher expression in purple leaf veins were mainly enriched in the flavonoid biosynthesis pathway. Integrating related genes and metabolites showed that the highly expressed genes of *ANS* (*BjuA004031*, *BjuB014115*, *BjuB044852,* and *BjuO009605*) and the excessive accumulation of dihydrokaempferol and dihydroquercetin contributed to the purple leaf veins by activating the synthetic pathways of pelargonidin-based anthocyanins and delphinidin-based anthocyanins. Meanwhile, “alpha-farnesene synthase activity” and “glucan endo-1, 3-beta-D-glucosidase activity” related to the adversity were mainly enriched in the serrated and lobed leaves, indicating that the environmental pressure was the dominant factor controlling the change in leaf shape. Overall, these results provided new insights into the regulation pathways for formation of purple leaf veins and leaf edge cracks, which could better accelerate the theoretical research on purple leaf vein color and leaf edge cracks in mustard.

## 1. Introduction

Mustard (*Brassica juncea* L.) is an annual herb that belongs to *Brassica* in the *Cruciferae* family that is cultivated and consumed mainly due to its edible stalks and leaves. In addition, it is an important oilseed and condiment crop [1]. Due to genetic variation, the cultivated species exhibit extensive morphological polymorphisms including the phenotypes of leaves, stems, roots, or seeds. These polymorphisms are the vital material basis for plant phenotypic research and directional breeding [2]. Among these polymorphisms, due to the important economic value, the study of leaf color and leaf shape has become the focus of researchers.

Leaf color is one of the easily recognizable morphological markers that influence consumer preference and market competitiveness. For leafy mustard cultivars, purple color of the leaf blade or leaf vein is a typical characteristic of great use in the study of germplasm resources. For example, purple leaves can be used as visible markers of plant transformation [3] and to rapidly identify seed purity in hybrid seed production at the seedling stage. Previous studies found that the formation of purple leaves is mainly related to the accumulation of anthocyanins [4]. Anthocyanins are natural food colorants that play important roles in the life activities of animals or plants, such as in antioxidant activities [5] and in resistance to biotic and abiotic stresses [6]. Various genes involved in the biosynthesis pathway of anthocyanin have been widely explored in model plants including *Arabidopsis thaliana* [7,8], *Zea mays* [9], *Oryza sativa* [10], and so on. The ternary complex of MYB-bHLH-WD40 has been shown to play an important role in anthocyanin accumulation. In *Arabidopsis*, overexpression of the R2R3-MYB transcription factors (*PAP1*, *PAP2*, *MYB113*, and *MYB114*) increased anthocyanin accumulation [7], while in cauliflower, the upregulated expression of *BoMYB2* and *BoTT8* genes led to anthocyanin accumulation, resulting in purple leaves [11]. In contrast, the silent expression of the *BoMYBL2-1* gene resulted in the establishment of purple *Brassica oleracea* leaves [12]. In *Brassica* species, several candidate genes or loci related to purple color have been identified, such as *BrEGL3.1* and *BrEGL3.2* in *zicaitai* (*B. rapa* L. ssp. *chinensis* var. *purpurea*) [13], *BoDRF* gene in Chinese kale (*Brassica oleracea* var. *alboglabra*) [14], and so on. However, the regulatory mechanism of anthocyanin accumulation that results in purple leaf in *Brassica* vegetables remains unknown. 

The diversity of leaf shape is one of the reasons for the diversity among species [15], and it affects the physiological functions of plants. Previous studies found that serrated or deeply cracked leaves increased the distance between leaves, which could increase heat dissipation and enhance disease defense [16]. The leaves of *Capsella rubella* have plasticity to temperature; the temperature is negatively related to the depth of leaf edge cracks, which means that the higher the temperature, the shallower the depth of cracks [17]. The difference in leaf shape in tomato also affects the flavor and quality of the fruits [18]. Leaf shape is not only regulated by genes [19,20,21], but is also influenced by the environment and hormones [22,23]. In *Arabidopsis thaliana*, the *CUP-SHAPED COTYLEDON1 (CUC1)*, *CUC2,* and *CUC3* genes regulate the formation of primitive shoot apex meristems and constrain the borders of cotyledons [24]. *CUC1* and *CUC2* are the target genes of small RNA *miR164*. The balance between *miR164* and *CUC2* determine the degree of leaf margin cracking [25]. The recessive mutation of *CUC2* leads to a decrease in the degree of leaf margin cracking, while the overexpression of *CUC2* leads to an increase in leaf margin cracking by destroying *miR164* [26]. Saddic found that the *LATE MERISTEM IDENTITY 1 (LMI1)* gene plays an important role in the formation of a single serrated leaf [27]. The *REDUCED COMPLEXITY (RCO)* gene could inhibit local leaf growth and change leaf shape via negative regulation with multiple cytokinins (CK) [28]. Moreover, Gupta et al. found that the leaf morphology of different sweet potato varieties varied greatly in different geographical locations, which indicated that the environment also affected the leaf shape [29]. In *Brassica*, some candidate genes related to leaf edge cracks were reported, such as *BnaA10g26320D* and *BnaA10g26330D* in *Brassica napus* [30,31] and *BrLMI1* in *Brassica napus* [32]. However, there are few studies that reported the genes associated with leaf edge cracks in *Brassica juncea*, especially at the transcriptome level. Therefore, the molecular mechanism of the formation of leaf edge cracks in *Brassica juncea* requires further study.

In this study, different types of mustard leaf samples (purple vein, green vein, different degrees of leaf marginal dehiscence) were collected. Through newly developed multi-omics technology, including RNA sequencing (RNA-Seq) and metabolic assays, the complex biosynthesis pathways that result in purple leaf veins and different degrees of leaf edge cracks were dissected in *Brassica juncea*. The results provided novel knowledge in the understanding of the formation of purple leaf veins and different degrees of leaf edge cracking in *Brassica juncea*, which could be a solid theoretical basis for the breeding application.

## 2. Results

### 2.1. Comparison of Morphological Phenotypes among Mustards

**Figure 1:**
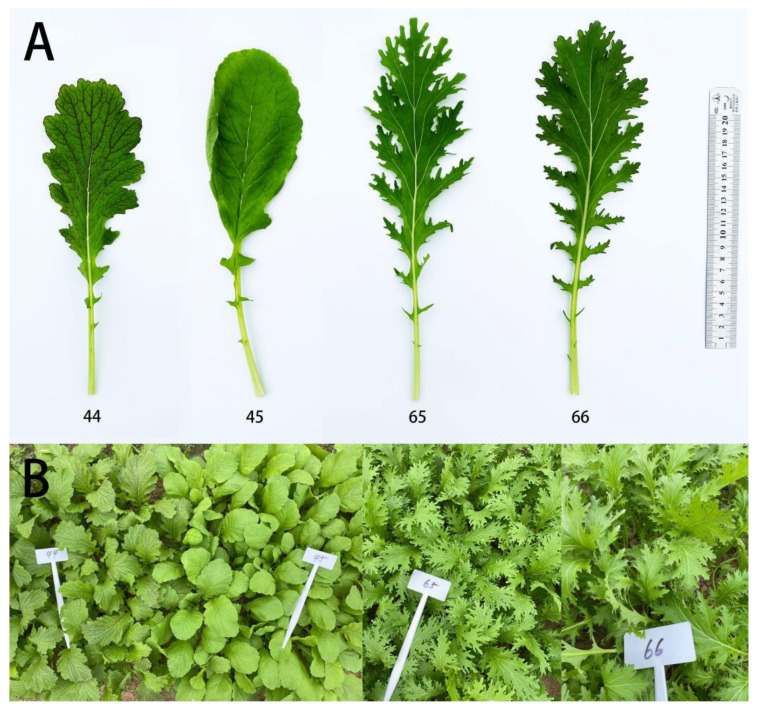
The morphological phenotypes of the four mustard cultivars. (**A**) The phenotype of each single leaf. (**B**) Overall plant phenotypes: 45 has green veins and an entire leaf; 44 shows purple veins and a serrated leaf; 65 shows green veins and a lobed leaf; 66 exhibits purple veins and a lobed leaf.

Significant differences were observed in the morphological phenotypes among 44, 45, 65, and 66, especially in the leaf color and type. As shown in Figure 1, the leaves of 44 and 66 exhibited purple veins compared with those of 45 and 65, which had green veins. Furthermore, when comparing the entire leaves of 45, 44, 65, and 66, each showed different degrees of leaf edge cracking: 44 exhibited a serrated leaf, while 65 and 66 showed a lobed leaf.

### 2.2. Identification of Differential Expression of Genes with RNA-Seq

**Figure 2:**
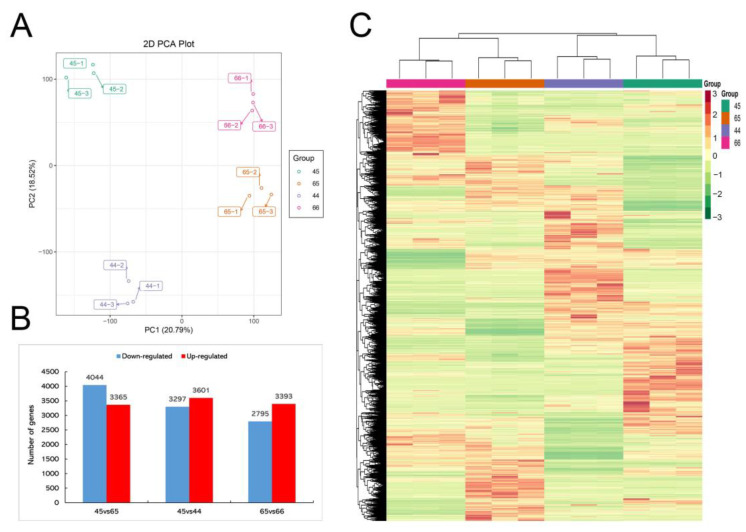
Multivariate statistical analysis of genes from four mustards. (**A**) PCA score plot of genes of the four mustard samples; the *x*-axis represents the first principal component and the *y*-axis represents the second principal component. (**B**) The numbers of DEGs in different sample combinations. (**C**) The hierarchical clustering analysis and heatmaps for DEGs.

A total of 48.69–58.05 million raw reads were obtained via Illumina sequencing of the mustard leaf samples. After removing reads containing adapters or poly-N and low-quality reads, clean reads were obtained with Q30 percentages of 90.47–92.77% and GC percentages of 47.37–47.86% (Table 1). The transcriptome data of different mustard leaf samples were separated using PCA; PC1 and PC2 were 20.79% and 18.52%, respectively (Figure 2A). One group of samples was clearly distinguished from the other three groups of samples and three repeated samples in each group were gathered together, indicating that the samples had good reproducibility and that there were significant differences in gene expression levels among different groups. There were 7409, 6898, and 6188 DEGs in the three comparison groups of 45 vs. 65, 45 vs. 44, and 65 vs. 66, respectively (Figure 2B). To clearly exhibit the differences among different groups, the FPKM values of DEGs among different groups were extracted for a hierarchical cluster analysis (Figure 2C). Those DEGs among different groups may contribute to the different phenotypes of mustard to a great extent.

### 2.3. K-Means Cluster and Enrichment Analyses of DEGs Associated with the Formation of Different Phenotypes

**Figure 3:**
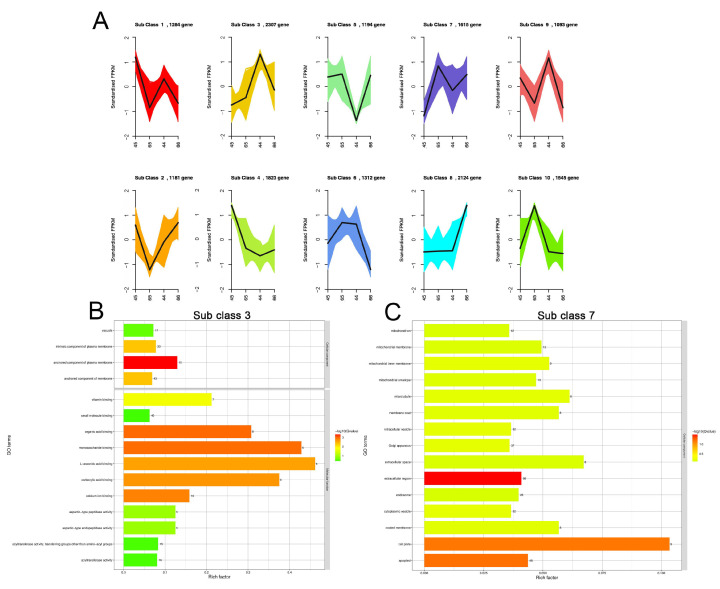
K-means cluster analysis and functional GO pathway classification of the DEGs. (**A**) Dynamics of DEGs among four samples. The bold black line represents the average pattern of all DEGs in each class, the different colored lines represent different classes, and each line represents the standardized FPK over three biological replicates. (**B**) GO-enriched analysis of DEGs in subclass 3. (**C**) GO-enriched analysis of DEGs in subclass 7.

Genes with similar expression patterns may play a similar function. To understand gene-expression patterns among different mustard leaf samples, the K-means clustering algorithm was used. The results of the K-means cluster analysis showed that the DEGs were divided into 10 subclasses. Combined with the phenotypes of different samples, subclasses 3 and 7 were selected for further study. The genes in subclasses 3 and 7 totaled 2307 and 1615, respectively (Table S1). Compared with the green vein samples (45 and 65), the genes in subclass 3 in the purple leaf vein samples (44 and 66) showed higher expression trends (Figure 3A), indicating that these genes may play a regulatory role in the formation of purple leaf veins. Similarly, in subclass 7, compared with the entire leaf sample (45), the genes in the serrated leaf sample (44) and lobed leaf samples (65 and 66) showed higher expression trends (Figure 3A), indicating that the high expression levels of these genes resulted in different degrees of leaf edge cracking.

To identify the functions of DEGs in subclasses 3 and 7, a Gene Ontology (GO)-enriched analysis was performed (Figure 3B,C). Based on the GO-enriched analysis, in subclass 3, the most significantly enriched molecular functions (MFs) were “L-ascorbic acid binding”, “calcium ion binding”, “acyltransferase activity”, and “small molecule binding”. The most significantly enriched cellular components (CCs) were “endoplasmic reticulum”, “intrinsic component of membrane”, and “cell periphery”. There was no biological process (BP) enriched. In subclass 7, only the CCs were significantly enriched, including “isomerase activity”, “extracellular region”, “cell plate”, “apoplast”, “extracellular space”, and so on. All of the GO enrichment results are shown in Tables S2 and S3. To further explore the biological pathways of formation of different mustard leaf samples, the DEGs in subclasses 3 and 7 were annotated based on the Kyoto Encyclopedia of Genes and Genomes (KEGG) database. The results showed that in subclass 3, the flavonoid biosynthesis, flavone and flavonol biosynthesis, and phenylalanine metabolism were the most significantly changed pathways (Table S4), which are the typical metabolic pathways that form plant color. In subclass 7, the pathways of carbohydrate metabolism, oxidative phosphorylation, energy metabolism, and citrate cycle (TCA cycle) were significantly enriched. In particular, the pathways enriched in environmental adaptation may also contribute to the change in leaf shape between 45 and the other three mustard leaf samples (44, 65, 66) (Table S5).

**Figure 4:**
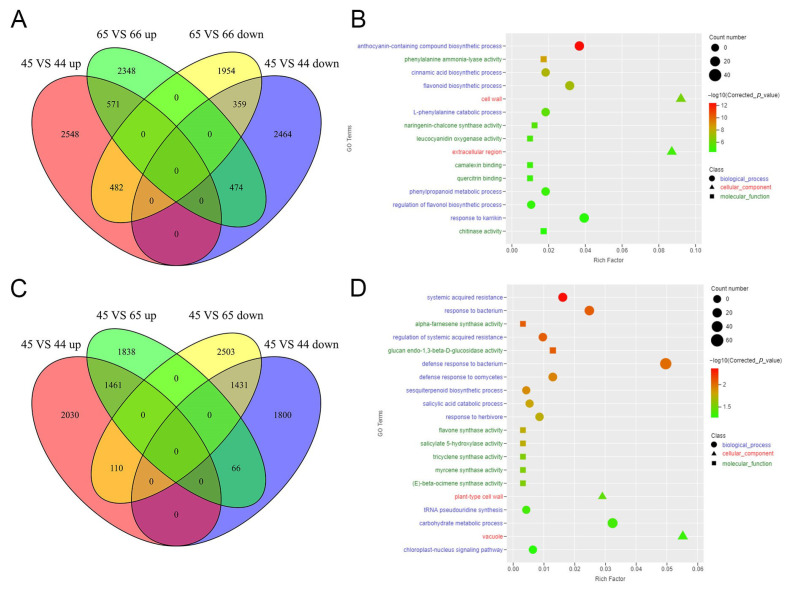
Statistics analysis of the DEGs. (**A**,**C**) Venn diagrams depicting the common and unique numbers of DEGs among the four mustard samples; (**B**,**D**) GO-enriched analysis of the common upregulated genes in purple-veined and edge-cracked leaves, respectively.

To further explore the key genes controlling leaf color, we compared and analyzed the DEGs among the four samples. We focused on the genes highly expressed in the 45 vs. 44 and 65 vs. 66 groups. As Figure 4A shows, 571 genes were highly expressed in 44 and 66 compared to 45 and 65, respectively. The GO-enriched analysis of the 571 genes showed that the most significantly enriched biological process was the anthocyanin-containing compound biosynthetic process (Figure 4B), which included 14 genes highly expressed in purple leaf veins. These results indicated that the enhancement of anthocyanin biosynthesis resulted in purple leaf veins. Similarly, for the leaf edge cracks in four samples, there were 1461 genes highly expressed in 44 and 65 compared with 45 (Figure 4C). The GO-enriched analysis showed that “systemic acquired resistance”, “alpha-farnesene synthase activity”, and “glucan endo-1,3-beta-D-glucosidase activity” were significantly enriched (Figure 4D), indicating that the serrated leaf and lobed leaf were selected by environmental pressure.

### 2.4. Differential Accumulation of Metabolites in Four Mustards

**Figure 5:**
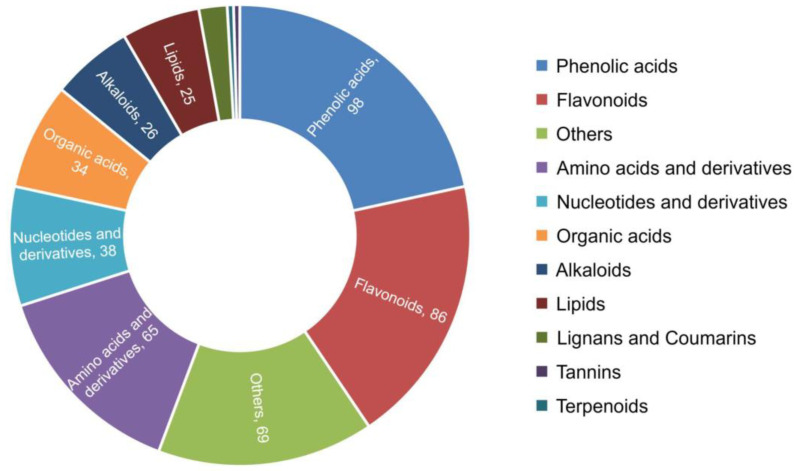
Pie chart of the classification of 454 metabolites.

Changes in plant phenotypes involve a variety of metabolites being synthesized and degraded. To recognize metabolite accumulation patterns on a global level, metabolome analyses of four mustard samples were performed using UPLC-MS/MS. A total of 454 metabolites were detected. These metabolites contained 98 phenolic acids, 86 flavonoids, 65 amino acids and derivatives, 38 nucleotides and derivatives, 34 organic acids, and so on (Figure 5). The PCA results showed that the first three principal components explained 27.63%, 21.65%, and 17.71% of the samples’ variance, respectively (Figure S1A). All the biological replicates in each group showed correlation coefficients above 0.9, indicating good reproducibility existed in the three biological replicates of each group (Figure S1B). A heat map of metabolites clearly distinguished the mustard samples with different phenotypes (Figure S1C). All results showed that the diversity of the metabolites was the reason for the formation of different phenotypes of mustards.

**Figure 6:**
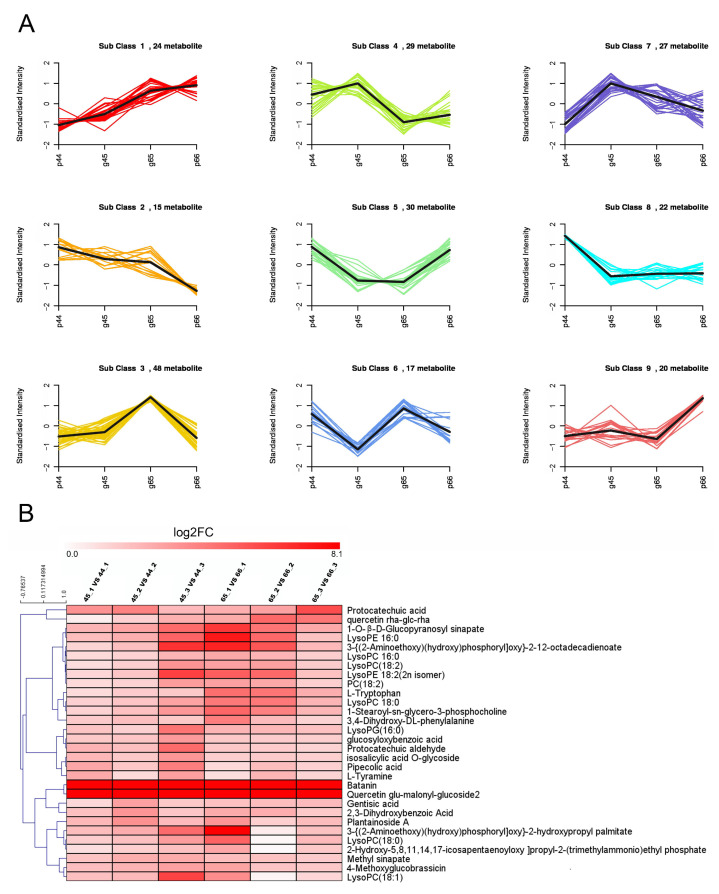
K-means analysis of differential metabolites. (**A**) Dynamics of metabolites for the four mustard samples. The bold black line represents the average pattern of all candidate metabolites in each class, the different colored lines represent different classes, and each line represents the standardized value of the metabolite over three biological replicates. (**B**) Hierarchical clustering heat map of 30 differential accumulation metabolites in subclass 5.

Based on the OPLS-DA results and using the criteria VIP > 1 and absolute log2FC > 1, 103 metabolites (49 downregulated and 54 upregulated) exhibited an altered abundance between the 45 and 44 samples (Figure S2A), 116 metabolites (66 downregulated and 50 upregulated) were significantly different between the 65 and 66 samples (Figure S2B), and 106 metabolites (47 downregulated and 59 upregulated) were significantly different between the 45 and 65 samples (Figure S2C). To further clarify the reasons for the formation of different leaf phenotypes, all the DAMs were analyzed using a K-means clustering algorithm. The results showed that a total of nine subclasses were obtained, among which the content of candidate metabolites in subclass 5 exhibited a higher accumulation in the 44 and 66 samples (purple leaf veins) compared with 45 and 65 (green veins) (Figure 6A). The metabolites in subclass 5 could be considered as potential metabolites of purple leaf veins. In subclass 5, the metabolites’ accumulation of 30 DAMs was displayed in the form of a heat map. The more metabolites that accumulated, the more closely related they were to the formation of purple leaf veins. As Figure 6B shows, quercetin glu-malonyl-glucoside2 and betanin were the most accumulated metabolites in the 44 and 66 samples compared with the 45 and 65 samples, respectively. Betanin is classified as an anthocyanidins and quercetin glu-malonyl-glucoside2 is an important flavonoid, and both of them are major pigments in the formation of plant leaf color.

To explore the vital candidate metabolites causing purple leaf veins and leaf edge cracks in mustard, Venn diagrams were drawn based on the DAMs of 45 vs. 44 and 65 vs. 66 as well as 45 vs. 44 and 45 vs. 65. The results indicated that 16 metabolites were highly accumulated in purple leaf vein samples (44 and 66) compared with green vein samples (45 and 65), and 5 metabolites were downregulated in purple leaf vein samples (Figure S3A; Table 2). This was consistent with the above results showing that the metabolites of quercetin glu-malonyl-glucoside2 and betanin were highly accumulated in purple leaf veins, indicating once again that these two metabolites were important for the change in the leaf vein color. In addition, one metabolite belonging to anthocyanins was downregulated in the two purple leaf vein samples. Regarding the leaf shape diversity, 15 metabolites were upregulated in the notched leaf samples (44 and 65); among these, the highest content of metabolites was alkaloids, followed by amino acids and derivatives. In addition, 14 metabolites were downregulated, and the highest content of metabolites was phenolic acid (Figure S3B; Table 3).

### 2.5. Conjoint Analysis of Genes and Metabolites Related to Purple Leaf Veins and Notched Leaves

**Figure 7:**
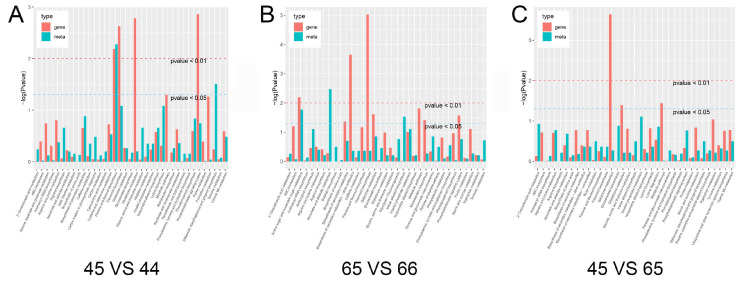
KEGG enrichment analysis of the DEGs and DAMs that were enriched in the same pathway. (**A**) KEGG enrichment analysis of the DEGs and DAMs in the 45 vs. 44 group. (**B**) KEGG enrichment analysis of the DEGs and DAMs in the 65 vs. 66 group. (**C**) KEGG enrichment analysis of the DEGs and DAMs in the 45 vs. 65 group. The abscissa represents the metabolic pathway, the red bars represent the enriched differential genes, and the blue bars represent the enriched differential metabolites (as expressed by log (*p*-value)). The higher the ordinate, the stronger the enrichment degree.

To reveal the relationship between key genes and metabolites associated with purple leaf veins and leaf edge cracks, the DEGs and DAMs in the 45 vs. 44, 65 vs. 66, and 45 vs. 65 groups were respectively mapped to the corresponding KEGG pathways. According to the results of the GO and KEGG enrichment analyses, the enrichment degrees of both the DEGs and DAMs in the pathways were shown with histograms (Figure 7). For instance, in the groups with purple leaf vein samples (45 vs. 44 and 65 vs. 66), the pathways for flavone, flavonol, and flavonoid biosynthesis were coenriched with higher credibility (Figure 7A,B). In addition, we analyzed the correlation between DEGs and DAMs in different groups and selected the results with a Pearson correlation coefficient greater than 0.8 (Table S6). The selected DEGs and DAMs were shown with correlation coefficients using a cluster heat map. The results showed that the pathway for flavonoid biosynthesis had a high enrichment degree for both differential genes and metabolites in the 45 vs. 44 and 65 vs. 66 groups (Figure S4A,B). In the 45 vs. 44 and 45 vs. 65 groups, for notched leaves, the pathways for phenolic acids and flavonoid biosynthesis had a high enrichment degree (Figure S4A,C).

### 2.6. Integrating Related Genes and Metabolites in the Flavonoid Biosynthesis Pathway Provides Insights into Purple Vein Formation

**Figure 8:**
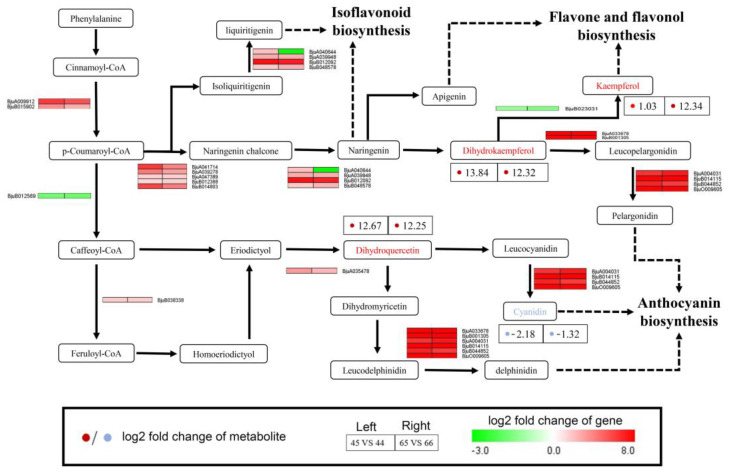
Detailed information on the DAMs and DEGs in the pathways for phenylpropanoid and flavonoid biosynthesis. The rectangle represents the DEGs (the red color represents high expression and the green color represents low expression). The dot represents the DAMs (the red color represents high expression and the blue color represents low expression).

Previous studies have shown that flavonoids and anthocyanins are important metabolites that lead to leaf color changes. To further clarify this point in this study, the DEGs and DAMs identified in both purple leaf vein samples (44 and 66) were selected to map to the flavonoid biosynthesis pathway. A total of 21 DEGs and four metabolites were screened (Figure 8). The results showed that the metabolites of dihydrokaempferol, dihydroquercetin, and kaempferol were highly accumulated in both 44 and 66. Dihydrokaempferol and kaempferol were the important metabolites in flavone and flavonol biosynthesis. In this pathway, the gene *BjuB023031* was downregulated, which regulated the synthesis of kaempferol. This was inconsistent with the accumulation of kaempferol. In addition, dihydrokaempferol also participated in anthocyanin biosynthesis. On this branch, the genes *BjuA033678*, *BjuB001305*, *BjuA004031*, *BjuB014115*, *BjuB044852*, and *Bjuo009605*, which were annotated as anthocyanin synthase oxygenase (*ANS*), showed high expression levels. Dihydroquercetin was a vital intermediate metabolite involved in anthocyanin biosynthesis. Following this metabolite, there were two branches for anthocyanin biosynthesis. On one branch, although the expression levels of genes associated with cyanidin biosynthesis were high, cyanidin involved in the pathway of anthocyanin biosynthesis presented as less accumulated in 44 and 66 compared with 45 and 65 separately. However, on the other branch, genes associated with delphinidin biosynthesis showed higher expression levels, while the contents of delphinidin in 44 and 66 were similar to those in 45 and 65, respectively.

**Figure 9:**
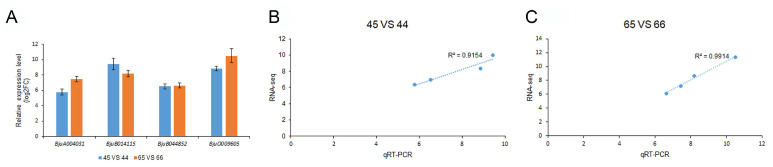
Gene expression and correlation analysis between qRT-PCR and RNA-Seq. (**A**) qRT-PCR analysis of 4 selected candidate genes. The blue color indicates the log2FC values in the comparison group of 45 vs. 44, the orange color indicates the log2FC values in the comparison group of 65 vs. 66. (**B**) Correlation analysis of gene expression between RNA-Seq and qRT-PCR in the comparison group of 45 vs. 44. (**C**) Correlation analysis of gene expression between RNA-Seq and qRT-PCR in the comparison group of 65 vs. 66.

To further validate the RNA-Seq expression profile data, four genes (*BjuA004031*, *BjuB014115*, *BjuB044852,* and *BjuO009605*) with high expression in the flavonoid biosynthesis pathway were selected for the qRT-PCR assays. The results showed that all four genes were consistent with respect to the expression profiles between the qRT-PCR analysis and the RNA-Seq data (Figure 9).

## 3. Discussion

It is well known that genes determine the phenotypes of organisms and that metabolomes are thought of as a “readout” of physiological states [33]. Therefore, exploring the association of gene-expression levels and metabolite abundances has attracted interest in recent years [34,35]. In the present study, we used four mustard samples that showed different phenotypes. Among these samples (44, 45, 65, and 66), 45 had green veins and an entire leaf, 44 showed purple veins and a serrated leaf, 65 showed green veins and a lobed leaf, and 66 exhibited purple veins and a lobed leaf (Figure 1). Using these samples, an integrated transcriptome and metabolome analysis was conducted to explain the variations in leaf color and leaf shape in mustard.

Flavonoids are the largest type of secondary metabolites and are the main compounds that determine the color of flowers, fruits, and leaves [36,37]. As important subclasses of flavonoids, flavonols and proanthocyanidins were proved to be the main pigment components in cucumber black thorn and orange peel [38], while the yellow peels of fruits such as lemons, oranges, limes, grapes, and tomatoes are often rich in flavanones [39,40]. Shi et al. showed that structurally modified anthocyanins and major potential copigmented flavonoids were the chemicals primarily responsible for the purple coloration of tea leaves [41]. In this study, dihydrokaempferol and kaempferol, which are important metabolites in the flavone synthesis pathway, were highly abundant in purple leaf veins compared with green veins (Figure 8). However, during the transformation from dihydrokaempferol to kaempferol, we identified only one differentially expressed regulatory gene (*BjuB023031*) that was downregulated in purple leaf veins. There may be some unknown regulatory mechanisms that lead to an increased accumulation of kaempferol. Excessive accumulation of kaempferol causes changes in the color of plant organs. Silva et al. (2020) found that high amounts of anthocyanins and other flavonoids such as rutin and kaempferol were responsible for the purple color of tomato [42]. In summary, our results showed that the excessive accumulation of dihydrokaempferol and kaempferol was involved in the formation of purple leaf veins in the 44 and 66 samples.

In addition, previous studies showed that the formation of purple leaves was mainly due to the accumulation of anthocyanins [43,44,45]; the pathway of anthocyanins in higher plants is well known, but the regulation of the biosynthetic mechanism in mustard remains unclear. Therefore, we focused on the regulatory pathway of anthocyanin synthesis. There were three anthocyanin synthesis pathways (Figure 8). It is well known that the metabolite of cyanidin is the most predominant anthocyanin found in nature [46]. In our study, there were four differentially expressed genes (*BjuA004031*, *BjuB014115*, *BjuB044852,* and *BjuO009605*) that regulated cyanidin synthesis. Although these four genes were consistently highly expressed in the purple leaf veins of the 44 and 66 samples, the accumulation of cyanidin was downregulated. Metabolites often present the final phenotype of plants, as the low accumulation of cyanidin results in low anthocyanin. Therefore, this branched pathway in anthocyanin biosynthesis was not the regulation pathway of purple leaf vein formation in mustard. Pelargonidin-based anthocyanins are another important branch for color characteristics [47]. Some scholars found that pelargonidin-based anthocyanins had a critical impact on the color and tinctorial strength of anthocyanin extracts [48]. Our results revealed that four genes were highly expressed; these were the vital genes in regulating the synthesis of pelargonidin. In this branch, from phenylalanine to anthocyanin, almost all differential genes were upregulated, indicating that the synthesis of pelargonidin-based anthocyanins was an important candidate regulatory pathway for purple leaf vein formation. The last pathway was the synthesis of delphinidin-based anthocyanins. Due to the large accumulation of delphinidin-related anthocyanins, the tea cultivar ‘Ziyan’ showed that a dark purple [49] and excessive accumulation of delphinidin-based anthocyanins resulted in novel bluer-colored *Chrysanthemums* [50]. In our results, genes and metabolites involved in the synthesis of delphinidin-based anthocyanins showed significant differences. Except for one gene (*BjuB012569*) regulating caffeoyl-CoA synthesis, which was downregulated, the other differentially expressed genes and proteins in this pathway were upregulated in purple-veined leaves. As a precursor metabolite of delphinidin synthesis, the dihydroquercetin metabolic level increased significantly in the two purple leaf vein samples. The above results indicated that the delphinidin-based anthocyanins also contributed to the purple leaf vein formation in the 44 and 66 samples. 

The shape of a plant leaf is also an obvious phenotype that not only restricts the yield of crops, but also affects stress resistance in plants. In this study, four mustard leaf samples showed three different phenotypes: 45 had an entire leaf, 44 showed a serrated leaf, and 65 and 66 showed a lobed leaf (Figure 1). The K-means cluster analysis showed genes in subclass 7 were highly expressed in the serrated and lobed leaves but showed low expression in the entire leaf. Moreover, the deeper the degree of leaf notching, the higher the expression levels of these genes (Figure 3). A GO-enriched analysis indicated that “isomerase activity”, “extracellular region”, “cell plate”, “apoplast”, and “extracellular space” were the most significantly enriched cellular components. Previous studies showed that the environment also affected leaf shape, with different geographical locations resulting in different leaf morphologies for potato [29]. In a similar finding, genes that were highly expressed in 44 and 65 compared with 45 were enriched in “systemic acquired resistance”, “alpha-farnesene synthase activity”, and “glucan endo-1,3-beta-D-glucosidase activity” (Figure 4D). All of them were related to adversity, indicating that the formation of serrated and lobed leaves was selected by environmental pressure [51].

## 4. Materials and Methods

### 4.1. Plant Materials

The purple leaf vein lines (44 and 66) and green leaf vein lines (45 and 65), which are self-bred lines bred by Anhui Science and Technology University, were planted in the experimental field at Anhui Science and Technology University (Fengyang, China). Among the four samples, 45 showed an entire leaf, 44 showed a serrated leaf, and 65 and 66 showed a lobed leaf. All the samples were collected from mature functional leaves, frozen in liquid nitrogen, and stored at −80 °C until use in RNA and metabolite extraction. All experiments were analyzed with three biological repeats.

### 4.2. RNA Sequencing and Data Analysis

The total RNA was extracted using a TaKaRa MiniBEST Plant RNA Extraction Kit (Code No.: 9769) following the manufacturer’s instructions. An Agilent 2100 RNA Nano 6000 Assay Kit (Agilent Technologies, Santa Clara, CA, USA) was used to detect the integrity and concentration of the RNA samples. Sequencing libraries were established using the NEBNext^®^ Ultra^TM^ RNA Library Prep Kit for Illumina^®^ (New England BioLabs, Inc., Ipswich, MA, USA) and sequenced on an Illumina Navoseq 6000 platform. Paired-end reads of these samples were generated.

By removing adapter sequences, ploy-N, and low-quality reads with Trimmomatic software (version = 0.39) [52], clean reads were obtained and checked with FastQC (http://www.bioinformatics.bbsrc.ac.uk/projects/fastqc, accessed on 5 January 2022) for the subsequent steps. Clean reads were mapped to the *B. juncea* reference genome [53] using TopHat v2.0.12, and the expression level of each gene was measured in fragments per kilobase of transcript per million fragments mapped (FPKM). The DESeq R package (1.18.0) was used to identify the differentially expressed genes (DEGs) with an adjusted false discovery rate (FDR) < 0.05 and |log2 FC (fold change)| ≥ 1.

Functional categorization of DEGs was analyzed using Blast2GO (version 2.6.4) (http://www.blast2go.org, accessed on 9 January 2022) [54]. Moreover, the DEGs were blasted against the online Kyoto Encyclopedia of Genes and Genomes (KEGG) database (http://geneontology.org, accessed on 9 January 2022) to retrieve their KOs and were subsequently mapped to pathways in KEGG [55]. GO enrichment of three ontologies (biological process, molecular function, and cellular component) and KEGG pathway enrichment analyses were applied based on Fisher’s exact test; the functional categories and pathways with a *p*-value < 0.05 were considered to be significant.

### 4.3. Metabolic Analysis

A total of 100 mg of powder ground from each freeze-dried leaf was weighted and extracted overnight at 4 °C with 0.6 mL of 70% aqueous methanol. Following centrifugation at 10,000× *g* for 10 min, the extracts were absorbed and filtrated. The purified samples were loaded into the UPLC-ESI-MS/MS system (UPLC, Shim-pack UFLC SHIMADZU CBM30A system; MS, Applied Biosystems 4500 Q TRAP). The parameters were set as follows: UPLC: column, Waters ACQUITY UPLC HSS T3 C18 (1.8 μm, 2.1 mm × 100 mm); the mobile phase consisted of solvent A (pure water with 0.04% acetic acid) and solvent B (acetonitrile with 0.04% acetic acid). Sample measurements were performed with a gradient program. The column oven was set to 40 °C. The injection volume was 4 μL. The effluent was alternatively connected to an ESI-triple quadrupole-linear ion trap (QTRAP)-MS. The ESI source operation parameters were set as follows: ion source, turbo spray; source temperature, 550 °C; ion spray voltage (IS), 5500 V (positive ion mode)/−4500 V (negative ion mode); ion sources gas I (GSI), gas II (GSII), and curtain gas (CUR) were set at 50, 60, and 30.0 psi, respectively; the collision gas (CAD) was high [56]. The raw files produced by the UPLC-ESI-MS/MS assays were analyzed with Analyst 1.6.3 software. Q3 was used for metabolite quantification, while Q1, Q3, RT (retention time), DP (declustering potential), and CE (collision energy) were used for metabolite identification.

A combination of fold change and the variable importance in projection (VIP) value of the OPLS-DA model was used to screen the differentially accumulated metabolites (DAMs) between groups. Significantly regulated metabolites between groups were determined using VIP > 1 and absolute log2FC (fold change) > 1. The identified metabolites were annotated using the KEGG compound database (http://www.kegg.jp/kegg/compound, accessed on 23 January 2022); annotated metabolites were then mapped to KEGG pathway database (http://www.kegg.jp/kegg/pathway.html, accessed on 23 January 2022). The pathways mapped with significantly regulated metabolites were then fed into a metabolite sets enrichment analysis (MSEA); their significances were determined using the hypergeometric test’s *p*-values.

### 4.4. Combined Analysis of DEGs and DAMs

The obtained DEGs and DAMs were mapped to the KEGG pathway for integration. The principal component analysis (PCA) diagram, hierarchical clustering analysis diagram, and Venn diagrams were drawn using the R software package. The Pearson correlation coefficients (PCCs) of the DEGs and the significantly regulated metabolites were calculated using the COR function in R. The genes and metabolites with a PCC > 0.8 in the KEGG pathway were used to establish the related network, which was visualized using Cytoscape software (version 3.7.1) [57].

### 4.5. qRT-PCR Validation of RNA-Seq Results

In total, 4 genes were selected for qRT-PCR analysis to examine the expression profiles of the RNA-Seq. The gene of BjActin was used as the internal control. The primers of the 4 genes for qRT-PCR were designed using Primer 6 software (Table S7). The cDNA was synthesized from the total RNA used for the RNA-Seq, and then the qRT-PCR analysis was performed on a Bio-Rad CFX96 using 2× SYBR green PCR master mix (Applied Biosystems) with three biological replicates. The relative expression of each gene was calculated using the 2^−ΔΔCT^ method.

### 4.6. Statistical Analysis

Statistical analysis was performed with SPSS software. All the data are shown with the average values of three repeated samples. Statistical significance was determined using the least significant difference test (*p* < 0.05).

## 5. Conclusions

In the present study, the main regulatory pathways of purple leaf veins and leaf edge cracks in mustard were revealed using combined transcriptomic and metabolomics analyses. Based on the differences in leaf color among the different mustard samples, the DEGs and DAMs with similar expression trends were identified using a K-means analysis. The highly expressed genes and metabolites in the purple leaf vein samples were mainly enriched in the flavonoid biosynthesis pathway. Integrating related genes and metabolites in the flavonoid biosynthesis pathway revealed that synthetic pathways of pelargonidin-based anthocyanins and delphinidin-based anthocyanins were the important pathways in regulating the formation of purple leaf veins in the 44 and 66 samples. For the formation of leaf edge cracks, “alpha-farnesene synthase activity” and “glucan endo-1,3-beta-D-glucosidase activity” related to adversity were mainly enriched in the serrated and lobed leaf samples, indicating that environmental pressure was the dominant factor in the formation of leaf edge cracks. In conclusion, our results provided new insights into the formation of purple leaf veins and leaf edge cracks in mustard, as well as a theoretical reference for further studies of leaf phenotypes.

## Figures and Tables

**Table 1:** Summary of RNA-Seq data from different types of mustard.

Samples	Raw Reads	Clean Reads	Q30 (%)	GC Content (%)	Unique Mapped
44-1	52,764,138	51,838,932	90.47	47.65	41,712,627 (80.47%)
44-2	57,898,070	56,684,510	92.36	47.71	46,074,374 (81.28%)
44-3	48,686,530	47,839,794	92.77	47.59	38,895,911 (81.30%)
45-1	59,411,784	58,048,268	92.23	47.75	47,126,857 (81.19%)
45-2	59,141,800	57,661,630	92.14	47.56	45,972,841 (79.73%)
45-3	52,856,618	51,892,220	92.01	47.64	41,742,258 (80.44%)
65-1	59,045,222	57,784,616	92.21	47.37	46,641,623 (80.72%)
65-2	52,647,580	51,702,172	91.46	47.61	41,756,075 (80.76%)
65-3	57,451,232	56,518,720	92.24	47.45	45,650,258 (80.77%)
66-1	54,027,590	53,162,134	92.64	47.86	42,963,193 (80.82%)
66-2	56,428,334	55,492,792	92.28	47.8	44,569,467 (80.32%)
66-3	51,296,644	50,462,792	92.09	47.62	40,610,737 (80.48%)

**Table 2:** The consistently significantly regulated metabolites in the 45 vs. 44 and 65 vs. 66 groups.

Metabolome ID	Compounds	Class I	Class II	Log2FC (45 vs. 44)	Log2FC (65 vs. 66)
CWJP002833	Kaempferol-di-O-malonyl-glucoside-O-malonyl-diglucoside	Flavonoids	Flavonols	13.84	1.64
Hmgp006596	Cyclobrassinin	Alkaloids	Plumerane	1.128	1.64
Hmyn007168	LysoPG(16:0)	Lipids	Glycerol ester	1.32	1.09
Lmmp002995	Quercetin glu-malonyl-glucoside2	Flavonoids	Flavonols	12.68	12.25
Lmyn001269	Kaempferol 3-O-β-D-sophoroside	Flavonoids	Flavonols	1.03	12.34
mws0126	1-Stearoyl-sn-glycero-3-phosphocholine	Lipids	LPC	1.156	1.72
mws0183	Protocatechuic acid	Flavonoids	Flavanols	1.63	1.70
mws0639	2,3-Dihydroxybenzoic acid	Organic acids	Organic acids	1.18	1.10
mws1138	Betanin	Alkaloids	Alkaloids	14.19	13.17
pmb0876	LysoPE 16:0	Lipids	LPE	1.68	1.74
pmb2936	Disinapoyl hexoside	Phenolic acids	Phenolic acids	1.76	12.55
pmb2940	1-O-β-D-Glucopyranosyl sinapate	Phenolic acids	Phenolic acids	1.66	1.97
pmd0136	LysoPC 18:0	Lipids	LPC	1.05	1.73
pmn001409	Plantainoside A	Phenolic acids	Phenolic acids	1.32	1.06
pmp001277	3-{(2-Aminoethoxy)(hydroxy)phosphoryl]oxy}-2-hydroxypropyl palmitate	Alkaloids	Alkaloids	1.69	1.68
pmp001286	LysoPC(18:0)	Lipids	LPC	1.15	1.67
Hmpp002612	Luteolin-7-O-β-D-gentiobioside	Flavonoids	Flavonoid	−2.19	−1.31
Lmbp002592	Kaempferol-3,7-di-O-β-D-glucopyranoside	Flavonoids	Flavonols	−2.34	−1.82
mws0170	Cyanidin chloride	Flavonoids	Anthocyanins	−2.26	−2.32
pme0256	Xanthine	Nucleotides and derivatives	Nucleotides and derivatives	−1.01	−1.09
pme0516	Inositol	Others	Saccharides and Alcohols	−1.66	−2.42

**Table 3:** The consistently significantly regulated metabolites in the 45 vs. 44 and 45 vs. 66 groups.

Metabolome ID	Compounds	Class I	Class II	Log2FC (45 vs. 44)	Log2FC (45 vs. 66)
Cmln000394	4-(Methylthio)-3-OH-butyl glucosinolate (glucoraphanin)	Others	Glucosinolates	1.41	1.37
CMLN000400	4-Methylsulfinyl-3-butenyl thioglucoside (glucoraphenin)	Others	Glucosinolates	1.83	1.33
GQ512003	Fer-agmatine	Alkaloids	Phenolamine	1.25	3.54
Hmgp006596	Cyclobrassinin	Alkaloids	Plumerane	1.13	1.86
mws0997	Petunidin 3-O-glucoside	Flavonoids	Anthocyanins	2.00	1.04
pmb0489	N-hexosyl-p-coumaroyl putrescine	Alkaloids	Phenolamine	3.60	2.75
pmb0490	N-p-coumaroyl putrescine	Alkaloids	Phenolamine	2.68	2.44
pmb0494	N-sinapoyl putrescine	Alkaloids	Phenolamine	2.17	1.98
pmb0496	N-feruloyl agmatine	Alkaloids	Phenolamine	1.15	3.72
pmb0608	Chrysoeriol O-malonylhexoside	Flavonoids	Flavonoid	1.21	1.10
pmb3081	Glucarate O-phosphoric acid	Others	Saccharides and alcohols	1.27	1.70
pme0008	L-citrulline	Amino acids and derivatives	Amino acids and derivatives	1.18	1.27
pme2596	4-Pyridoxic acid	Others	Vitamin	1.26	1.30
pme3388	H-homoArg-OH	Amino acids and derivatives	Amino acids and derivatives	2.30	1.84
Rfmb319	Pipecolic acid	Amino acids and derivatives	Amino acids and derivatives	1.03	1.56
Cmyn001733	1-Methylpropyl glucosinolate	Others	Glucosinolates	−4.29406	−3.09
CWJP002007	Kaempferol-3-O-feruloyl-sophoroside-7-oglucoside	Flavonoids	Flavonols	−1.57628	−1.29
Hmbn002228	kaeperferol-3-O-[2-O-(6-O-E-feruloyl)-β-D-glucopyranosyl]-β-galactopyranoside	Others	Others	−1.14448	−2.39
Lmhn002423	vnilloylmalic acid	Phenolic acids	Phenolic acids	−4.00812	−2.29
Lnrp102522	Kaempferol glc-glc-rha	Flavonoids	Flavonols	−3.30626	−1.23
mws1212	Methyl ferulate	Phenolic acids	Phenolic acids	−1.32806	−1.24
mws1550	S-allyl-L-cysteine	Amino acids and derivatives	Amino acids and derivatives	−1.65983	−1.36
pmb0382	O-feruloyl 4-hydroxylcoumarin	Lignans and Coumarins	Coumarins	−2.10723	−3.09
pmb2620	3,4-Dimethoxycinnamic acid	Phenolic acids	Phenolic acids	−1.35293	−1.09
pmb3072	3-O-p-coumaroyl shikimic acid O-hexoside	Phenolic acids	Phenolic acids	−1.81168	−2.06
pme2914	3-Hydroxy-3-methylpentane-1,5-dioic acid	Amino acids and derivatives	Amino acids and derivatives	−1.70006	−1.99
pme3382	N-acetylthreonine	Amino acids and derivatives	Amino acids and derivatives	−1.98759	−1.27
Zmhn001375	Vanillic acid-C-glucoside	Phenolic acids	Phenolic acids	−1.91953	−1.30
Zmhn001883	Vanillic acid glycoside	Phenolic acids	Phenolic acids	−1.64013	−1.12

## Data Availability

The raw data from the comparative transcriptome analysis were deposited into the Sequence Read Archive (https://www.ncbi.nlm.nih.gov/sra) under accession number PRJNA800112.

## Supplementary Materials

The following are available online at https://www.mdpi.com/article/10.3390/plants11172229/s1, Figure S1: The analysis results of the metabolic profiles obtained by UPLC-MS/MS. (A) Principal component analysis (PCA) score plot of all metabolites in 12 samples. The samples with the same color represent three biological replications of the same sample. (B) Correlation analysis of metabolome data of four samples. (C) The hierarchical clustering analysis and heatmaps of differential accumulation metabolites; Figure S2: Volcano map of differential metabolite. Each point in the volcanic map represents a metabolite, the x-coordinate represents the logarithm of the fold change of a metabolite in the two samples, and the y-coordinate represents the VIP value; Figure S3: The Venn diagram depicts the common and unique number of differential accumulation metabolites among the four groups of samples; Figure S4: Correlation coefficient cluster heat map of differentially accumulated metabolites and differentially expressed genes; Table S1: The information of DEGs in different subclasses; Table S2: GO analysis of DEGs in subclass 3; Table S3: GO analysis of DEGs in subclass 7; Table S4: KEGG analysis of DEGs in subclass 3; Table S5: KEGG analysis of DEGs in subclass 7; Table S6: Genes and metabolites with the Pearson correlation coefficient greater than 0.8 in the 45 vs. 44 group; Table S7: List of primers used in quantitative RT-PCR. All primers are designed by Primer 6.
