# Supplementary figures and images for: Integrated Analysis of Transcriptome and Metabolome Reveals New Insights into the Formation of Purple Leaf Veins and Leaf Edge Cracks in Brassica juncea
Source: Plants (Basel). 2022 Aug 28;11(17):2229. doi: 10.3390/plants11172229 (PMC9460116; doi:10.3390/plants11172229)

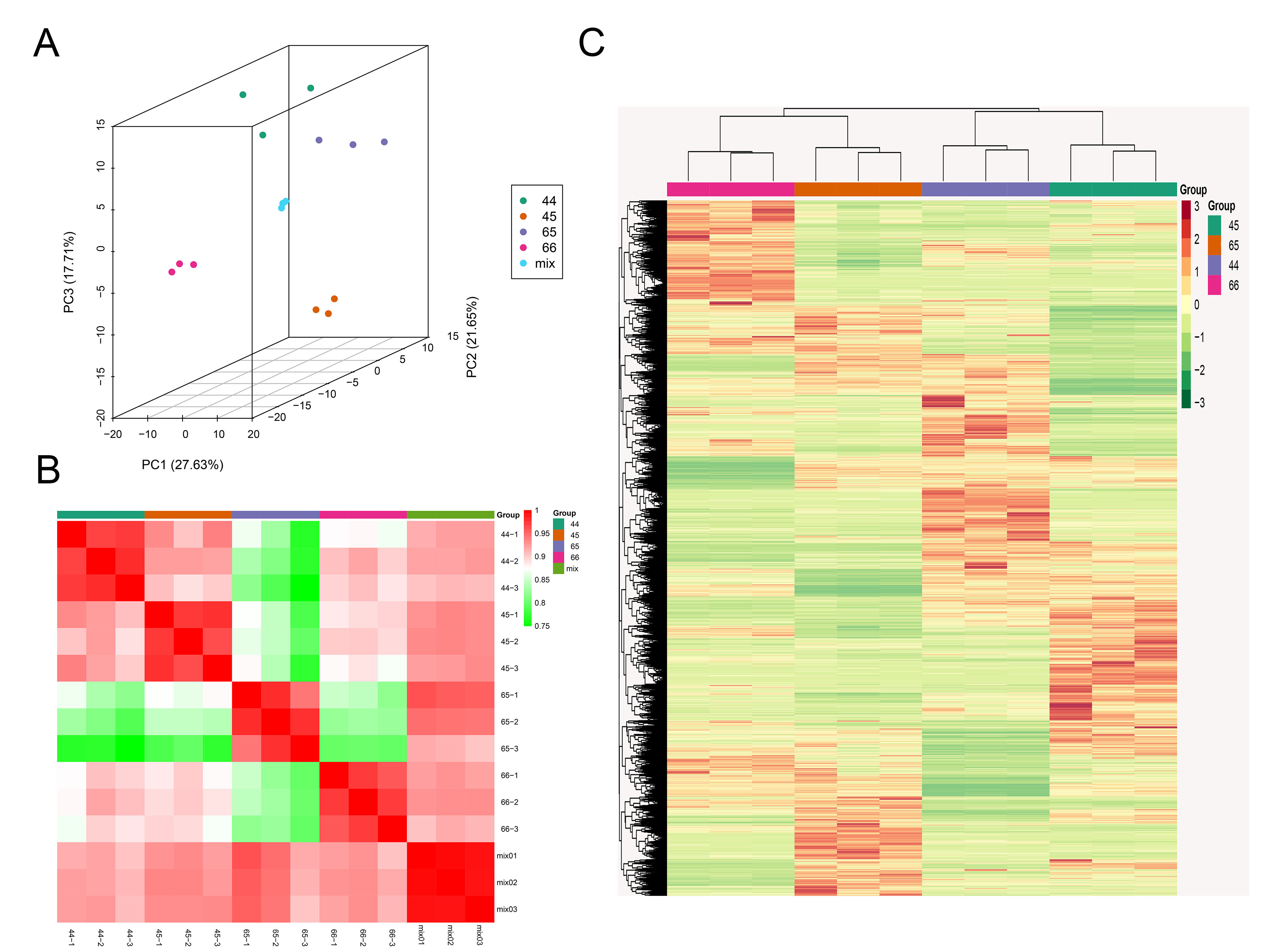

Supplement: Supplementary file 1 [file plants-11-02229-s001.zip › Supplementary figures/Figure S1.jpg]

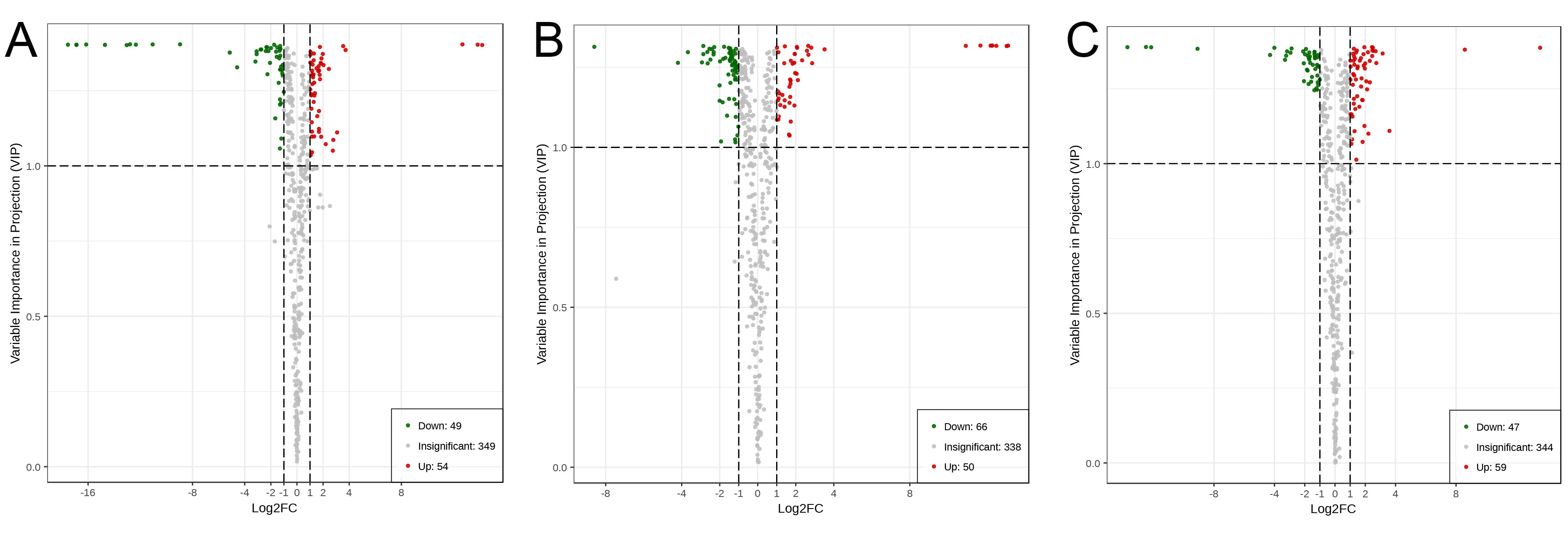

Supplement: Supplementary file 1 [file plants-11-02229-s001.zip › Supplementary figures/Figure S2.jpg]

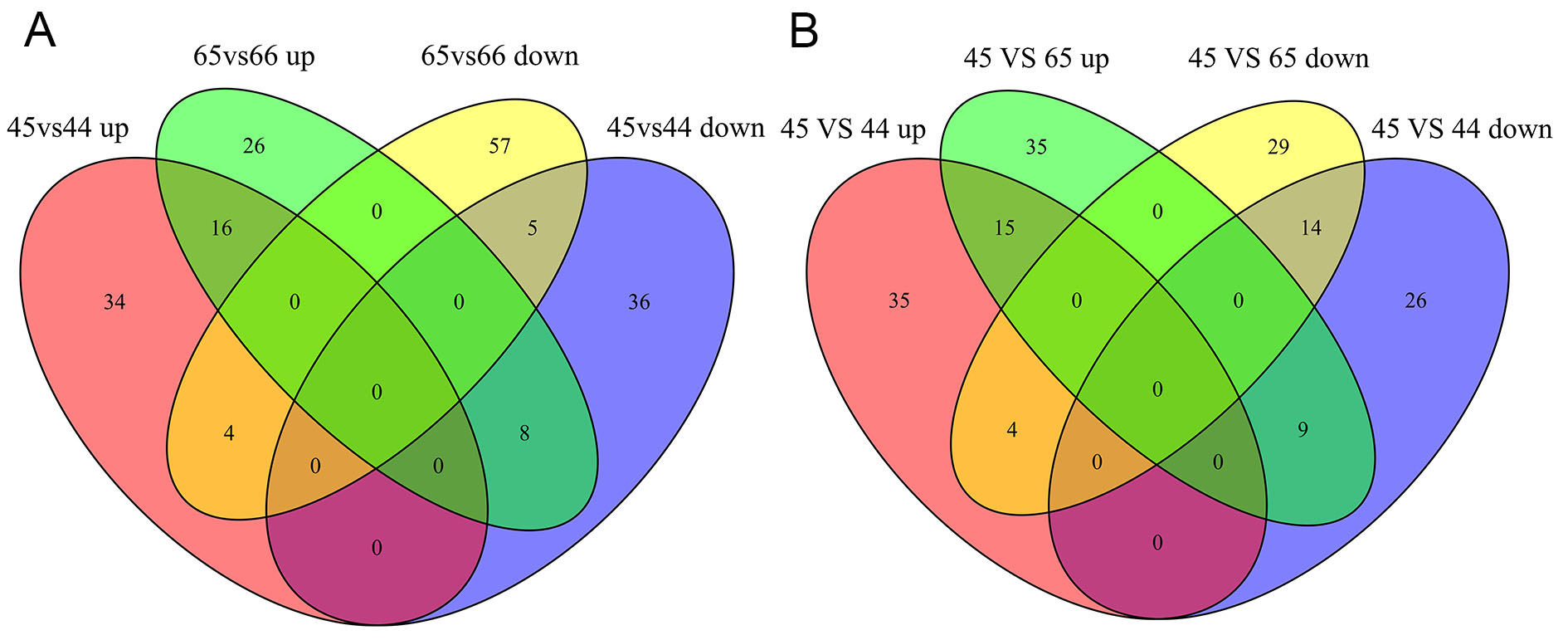

Supplement: Supplementary file 1 [file plants-11-02229-s001.zip › Supplementary figures/Figure S3.jpg]

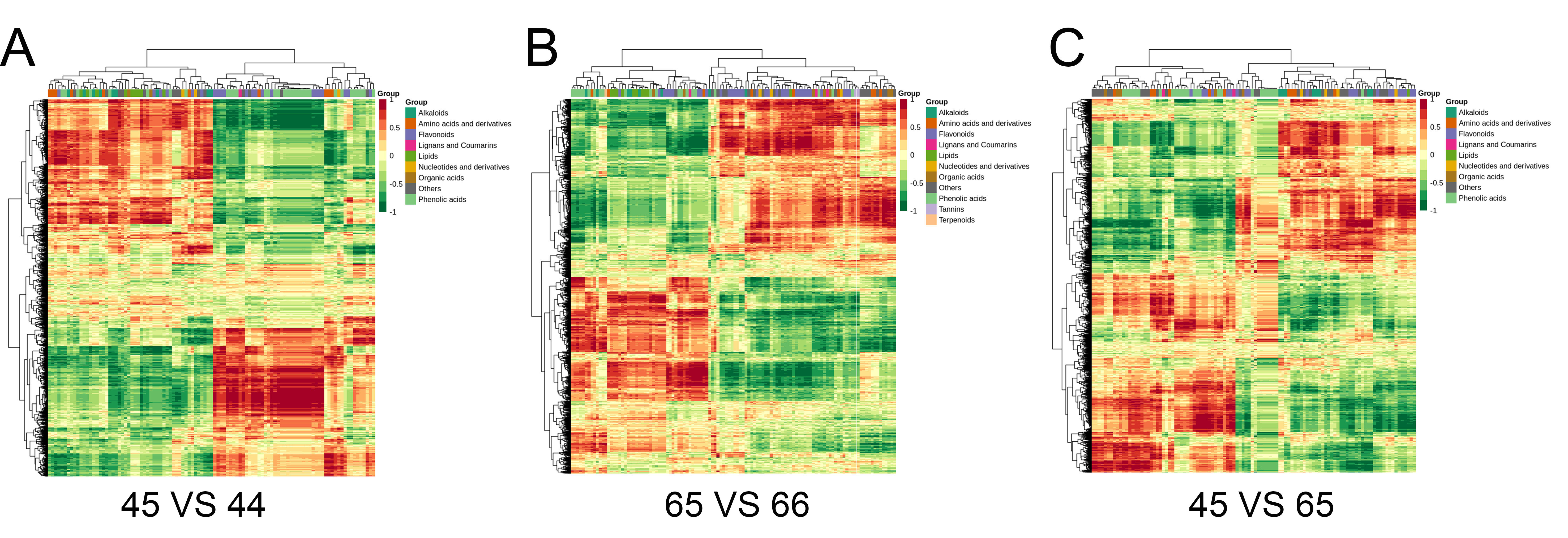

Supplement: Supplementary file 1 [file plants-11-02229-s001.zip › Supplementary figures/Figure S4.jpg]
